# Assessment of setup uncertainties for various tumor sites when using daily CBCT for more than 2200 VMAT treatments

**DOI:** 10.1120/jacmp.v15i2.4418

**Published:** 2014-03-06

**Authors:** Young‐Kee Oh, Jong‐Geun Baek, Ok‐Bae Kim, Jin‐Hee Kim

**Affiliations:** ^1^ Department of Radiation Oncology Keimyung University Dongsan Medical Center Daegu Republic of Korea; ^2^ Department of Physics Yeungnam University Kyongsan Republic of Korea

**Keywords:** systematic error, random error, daily CBCT, PTV margin

## Abstract

The aim of this study was assess the patient setup errors for various tumor sites based on clinical data from a sufficient number of treatments with volumetric‐modulated arc therapy (VMAT) using daily pretreatment CBCT imaging guidance. In addition, we calculated and compared the planning target volume (PTV) margins for all disease sites based on an analysis of specific systematic and random errors in our institution. All patients underwent pretreatment kV‐CBCT imaging. The various tumor sites were divided into four categories; 21 brain (438 fractions), 35 head‐and‐neck tumors (H&N, 933 fractions), 19 thorax and abdomen tumors (T&A, 313 fractions), and 17 prostate cancer tumors (546 fractions). Overall distributions of setup corrections in all directions, frequencies of 3D vector lengths, institution‐specific setup error, and PTV margins were analyzed. The longitudinal distribution for the T&A site represented an asymmetric offset in the negative direction. Rotational distributions were comparable for all treatment sites, and the prostate site had the narrowest distribution of $\leq \pm 2^{\circ }$. The cumulative frequencies of 3D vector length of ≥ 7 mm were rare for brain lesions and H&N, but more common for T&A and prostate lesions at 25.6% and 12.1%, respectively. The overall mean error for all treatment sites were within ±1 mm and $\pm 0.1^{\circ }$, with the exception of the T&A site, which had overall mean error of 2 mm in the negative longitudinal direction. The largest magnitude of systematic error and random error for the brain lesions and H&N was 1.4 mm in the translational directions, and 3.3 mm for T&A and prostate lesions. The PTV margins required in this analysis are ≤ 4 mm for the brain lesions and H&N in all translational directions, but ranged from 4 to 10 mm for T&A and prostate lesions. Analysis of each institution's specific setup errors using daily CBCT is essential for determining PTV margins and reducing setup uncertainties, because setup errors vary according to each immobilization system and patient.

PACS number: 87.55.km

## I. INTRODUCTION

Intensity‐modulated radiation therapy (IMRT) can deliver highly conformal radiation to the target, whilst sparing adjacent critical organs and other normal tissues.^1^, ^2^, ^3^, ^4^, ^5^, ^6^, ^7^ Increasingly sharp dose gradients generated by IMRT require more accurate target localization and precise patient positioning because small setup errors can compromise target coverage and increase the dose to organs at risk.^8^, ^9^, ^10^, ^11^, ^12^ Therefore, high geometric accuracy is particularly important for the clinical application of IMRT.

Image‐guidance systems are now commonly used to minimize setup uncertainties^13^, ^14^, ^15^, ^16^, ^17^, ^18^, ^19^, ^20^ and have provided highly effective for quantifying patient setup errors and correcting target localization^20^, ^21^ A reduction in setup uncertainties would allow for a reduction in treatment margins, leading to dose escalation and an improvement in local control at the tumor site^22^, ^23^, ^24^, ^25^, ^26^

Cone‐beam computed tomography (CBCT) is an especially useful image‐guidance modality for IMRT because it provides excellent visualization of the target with three‐dimensional (3D) imaging and high‐resolution, soft‐tissue information^27^, ^28^, ^29^

However, acquisition of CBCT images is a time‐consuming process compared to the acquisition of conventional, two‐dimensional (2D) portal imaging scans, and the total time needed for IMRT treatment including setup, pretreatment CBCT, repositioning, verification, and beam delivery is approximately 60 minutes.^(30)^ The prolonged treatment time may increase patient setup errors and intrafractional variations^(31)^

With the advent of volumetric‐modulated arc therapy (VMAT), these problems could be overcome. VMAT technology is a novel extension of IMRT, capable of delivering highly conformal dose distribution over a much shorter beam‐on time and requiring fewer monitor units than IMRT by the modulation of gantry rotation speed, multileaf collimator motion, dose rate, and the number of arcs.^32^, ^33^, ^34^, ^35^, ^36^ In addition, a single rotation of the gantry takes less than 2 minutes at the maximum gantry rotation speed.^(32)^ These improvements can significantly reduce the treatment time, thereby potentially reducing both the number of random daily setup errors in positioning and variations in patient anatomy during treatment.

Multiple studies have addressed the setup uncertainties associated with CBCT imaging guidance in IMRT, although only a few studies have included clinical data on the setup errors at various tumor sites using daily CBCT imaging guidance and the same immobilization system for each treatment site. Previous studies have addressed the setup uncertainties for various disease sites using CBCT imaging. However, the setup errors in one of these studies^(37)^ were analyzed with multiple setup positions (supine, prone, head first, feet first) and different immobilization systems (belly board or no immobilization device) for the same disease site. Moreover, the clinical data in another of these studies^(38)^ included both CBCT imaging and 2D electronic portal images and involved only 24 patients. Therefore, in order to examine the setup errors more accurately, the same immobilization system should be applied for each treatment site and more patients should be studied.

The aim of this study was to assess the patient setup errors for various tumor sites based on clinical data from a sufficient number of treatments using daily pretreatment CBCT imaging guidance. In addition, we calculated and compared the planning target volume (PTV) margins for all disease sites based on an analysis of specific systematic and random errors in our institution.

## II. MATERIALS AND METHODS

### A. Selected patient data

Between January 2011 and July 2012, more than 110 patients were treated with VMAT via RapidArc (Varian Medical Systems, Palo Alto, CA) in this institution, all of whom underwent pretreatment kV‐CBCT imaging. The various tumor sites were divided into four categories, and each site was treated using a site‐specific immobilization device. The anatomic treatment sites consisted of 21 brain tumors (438 fractions), 35 head‐and‐neck tumors (H&N, 933 fractions), 19 thorax and abdomen tumors (T&A, 313 fractions), and 17 prostate cancer tumors (546 fractions). Although several cases involved the pelvic region, including pelvic bone metastases and cervical cancer, they were not included in our study because of the different setup direction used — for example, prone position or a feet‐first direction. They also utilized different immobilization devices, such as the vacuum mold system. Patients undergoing stereotactic body radiation therapy (SBRT) were also excluded from this analysis because they required less than 4 treatment fractions.

### B. Patient immobilization and initial setup

**Figure 1:**
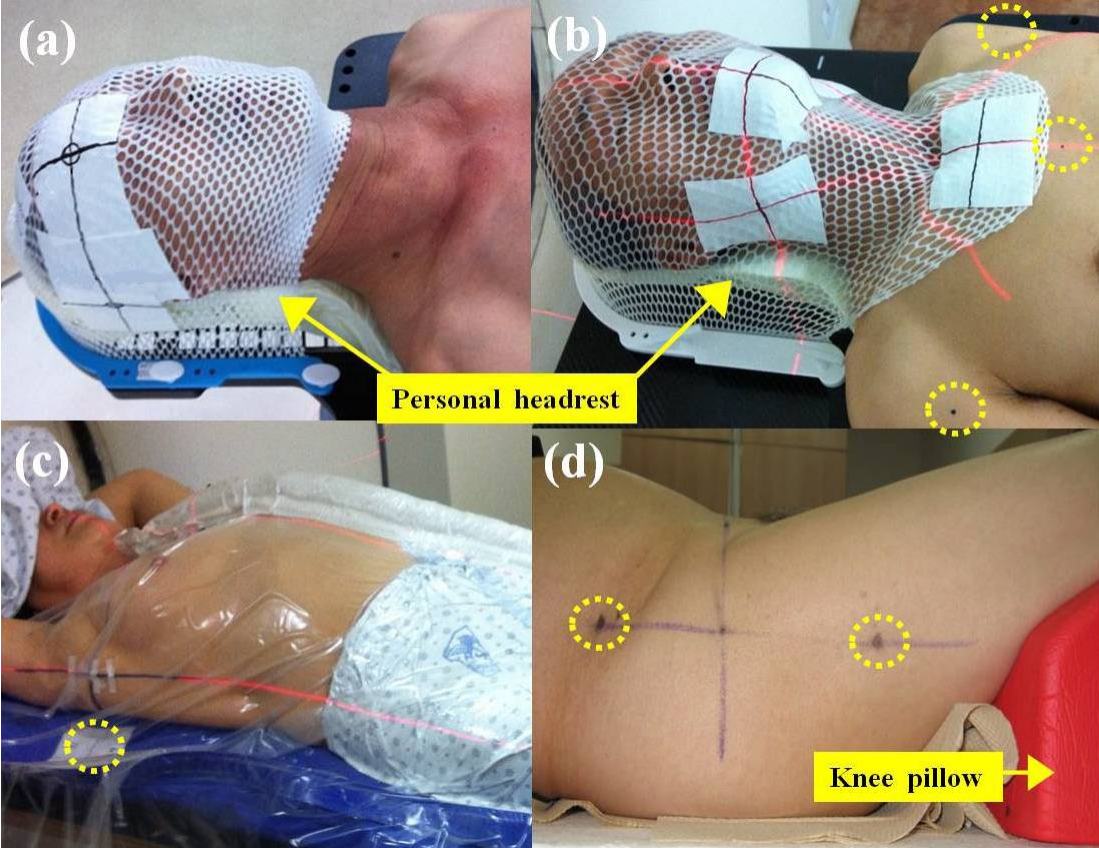
The immobilization systems: brain (a), H&N (b), T&A (c), prostate (d). Thermoplastic fixation mask and personal designed headrest are utilized for brain and H&N sites; double vacuum device is applied to T&A sites; knee pillow is used for prostate site. The dashed circles indicate additional straightening markers to assist alignment in all directions. H&N = head and neck, T&A = thorax and abdomen.

The immobilization systems for each treatment site are shown in Fig. 1. Patients with brain and H&N were immobilized using a thermoplastic fixation mask and an individually designed headrest. Patients with T&A were immobilized using the BodyFix dual vacuum system (Medical Intelligence, Schwabmunchen, Germany), which is a full‐body vacuum mold. Prostate cancer patients were only immobilized using knee pillow in the supine position, without any additional immobilization. An endorectal balloon was inserted into the rectum of all prostate cancer patients and filled with 60 mL of air for the computed tomography (CT) scan and each treatment fraction. In addition, these patients were instructed to have a full bladder for the simulation and daily treatment. The external markers for patients with brain lesions and H&N were placed on the surface of the mask along the setup laser line, and patients with H&N had additional three skin tattoos — one each was placed on each shoulder, and the third anterior tattoo was placed farther along the patient's superior/inferior axisto be use for straightening alignment. Patients with T&A had three skin tattoos and two lateral alignment markers placed on the evacuated cushion, prostate cancer patients had three skin tattoos and two lateral tattoos farther along and on both sides of the setup laser line for more accurate alignment.

### C. Image acquisition and CBCT to planning CT image registration

All patients underwent CT using a Somatom Emotion 6 helical CT scanner (Siemens Medical Systems, Erlangen, Germany) with a 3 mm slice thickness for all anatomic sites. Patients with T&A underwent additional CT procedures during the inhalation and exhalation phases, to assess internal organ motion during respiration. The resulting CT images were transferred to the Eclipse treatment planning system (Varian Medical Systems). The gross tumor volume (GTV) of each patient was determined on the basis of positron emission tomography (PET) CT images or magnetic resonance imaging (MRI) scans by comparing them with the original CT images. For every treatment fraction, the patients were immobilized and positioned according to the markers or tattoos by using the in‐room setup lasers. Thereafter, kV‐CBCT images were acquired using the gantry‐mounted on‐board imager (OBI) (Varian Medical Systems). As shown in Table 1, in our institution, the current OBI systems have six available acquisition modes of kV‐CBCT in the scanning protocol. The low‐dose mode was used to acquire the CBCT images in order to rduce the radiation exposure dose of every CBCT fraction except that delivered to the pelvis, as there was no suitable low‐dose mode available. The acquisition time is approximately 30 seconds for the low‐dose head mode with a gantry rotation of 200°, and approximately 1.5 minutes for both low‐dose thorax and pelvis mode with gantry rotation of 360°. All acquired kV‐CBCT images have a sufficient scan length to cover the full target of each anatomic site with a 3 mm slice thickness. Quality assurance for accuracy of OBI alignment was performed with threshold within ±1 mm once a month in our institution.

The registration procedure between the acquired CBCT images and planning CT images was performed for all treatment sites according to the bony anatomy and soft tissue distribution. The registration for the brain lesions was based on the bony anatomy, and for the H&N, this was based on both bony and soft tissue anatomy. Matching for T&A was based on different types of anatomy comparisons: the rigid bony anatomy, which is usually the vertebral column; visualized target volume within the PTV; and adjacent landmarks such as the carina or bronchus. For the prostate site, the alignment was based on the discernible tumor location with respect to the shape of endorectal balloon and bladder filling, as well as the pelvic bony anatomy.

The registration was first performed automatically by the OBI registration system and was then manually checked by therapists and confirmed by treating physicians to ensure that the matching was accurate. Patient setup and target localization corrections were use by the automatic adjustment couch system in three translational directions (lateral, longitudinal, vertical) and the rotational (yaw) direction.

**Table 1:** Parameters for each specified kV‐CBCT image acquisition mode

	*Low‐Dose Head*	*Low‐Dose Thorax*	*Standard‐Pelvis*	*High‐Quality Dose Head*	*Pelvis Head*	*Spotlight*
X‐ray voltage (kVp)	100	110	125	100	100	125
X‐ray current (mA)	10	20	80	20	80	80
X‐ray millisecond (ms)	20	20	13	20	25	25
Scan fan type	Full fan	Half fan	Half fan	Full fan	Full fan	Full fan
Bow‐tie filter	Full	Half	Half	Full	Full	Half
Gantry rotation range (°)	200	360	360	200	200	200

### D. Statistical analysis and PTV margin calculation

Setup correction data for all treatment sites were collected into registration software as Offline Review (Varian Medical System). Clinical data were divided into four categories: overall distributions of setup corrections of all fractions, frequencies of 3D vector lengths, institution‐specific setup errors, and PTV margins. Frequencies of 3D vector lengths were quantified to assess how often translational corrections occurred and the magnitudes were calculated using the following formula:

(1) $$\sqrt{x^{2}+y^{2}+z^{2}}$$

where *x, y*, and *z* represent the lateral, longitudinal, and vertical setup correction, respectively. Institution‐specific setup errors consisted of three certain errors define by Remeijer et al.:^(39)^ the overall mean error (M), the systematic error (Σ), and the random error (σ) define as follows:

(2) $$m_{p}=\sum_{f=1}^{F_{P}}\frac{x_{pf}}{F_{P}}$$

(3) $$N=\sum_{p=1}^{p}F_{P}$$

(4) $$M=\frac{1}{N}\sum_{p=1}^{P}\sum_{f=1}^{F_{P}}x_{pf}$$

(5) $$\sum =\sqrt{\frac{1}{N(P-1)}\sum_{p=1}^{p}F_{P}{\left(m_{p}-M\right)}^{2}}$$

(6) $$\sigma =\sqrt{\frac{1}{N-P}\sum_{p=1}^{p}\sum_{f=1}^{F_{p}}{\left(x_{pf}-m_{p}\right)}^{2}}$$

In these equations, $F_{p}$ is the measured fractions for each patient p, *f* is the number of fractions used to treat a patient, $x_{pf}$ is the measurement of a setup error for each fraction, $m_{p}$ is the average patient setup error, and *N* is the total number of measured fractions. The *M* statistic is the average of all setup corrections measured for all patients and is small (close to zero), unless significant imprecision was present in the equipment or procedures. The Σ statistic is define as the standard deviation (SD) of the means per patient and is a measure of how reproducibly the treatment setup is performed. The σ statistic is the root mean square of the individual SD and represents the magnitude of the patient‐specific random error.

Several PTV margin recipes using Σ and σ have been reported to adapt the appropriate PTV margin.^40^, ^41^ In this study, the general margin recipe, which can be applied to all tumor types, was used for all treatment sites. As a result, the commonly used margin recipe from van Herk^(40)^ was applied to all treatment sites in the present study. Therefore, the PTV margin was calculated using this formula:

(7) 2.5∑+0.7σ

## III. RESULTS

**Figure 2:**
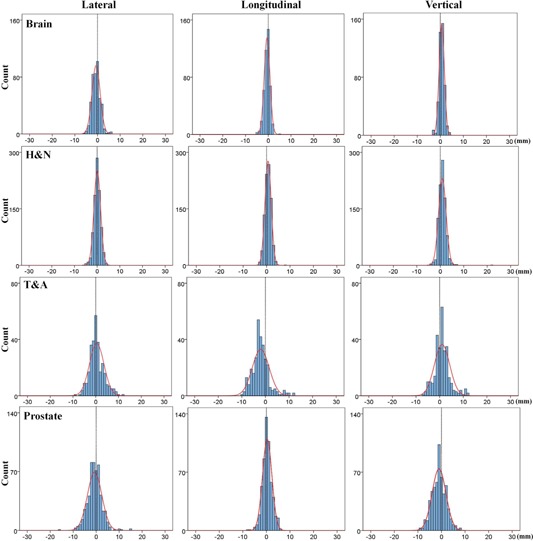
Overall distributions of translational shift in each treatment site. The distributions are narrow for brain, H&N, and prostate, whereas the longitudinal distribution for T&A represents asymmetric offset toward the negative direction. H&N = head and neck, T&A = thorax and abdomen.

**Figure 3:**
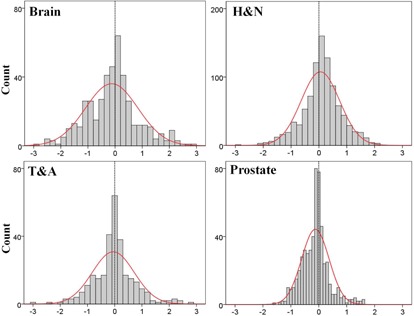
Overall distributions of rotational shift in each treatment sites (°). All distributions do not exceed $\pm 3^{\circ }$; the prostate has the narrowest distribution. H&N = head and neck, T&A = thorax and abdomen.

The overall distributions of translational setup corrections for all treatment sites in this study are shown in Fig. 2. The distributions were narrow and did not exceeded ±10 mm for brain lesions and H&N, whereas distributions of the T&A and prostate lesions were broad, with the exception of the longitudinal direction for the prostate site. The longitudinal distribution for the T&A site represented an asymmetric offset in the negative direction. Rotational setup correction distributions are shown in Fig. 3. The distributions were comparable for all treatment sites, and the prostate site had the narrowest distribution of $\leq \pm 2^{\circ }$.

**Figure 4:**
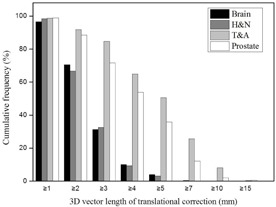
Cumulative frequencies of 3D vector lengths for translational setup errors. The frequencies of ≥ 7 mm are not observed for brain and H&N, whereas the values of ≥ 10 mm represent T&A and prostate. H&N = head and neck, T&A = thorax and abdomen.

**Figure 5:**
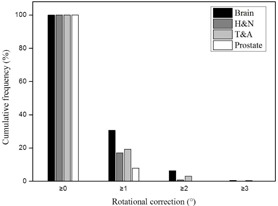
Cumulative frequencies of rotational setup errors. All frequencies are comparable regardless of treatment site; brain has the highest value of ≥ 1°. H&N = head and neck, T&A = thorax and abdomen.

The cumulative frequencies of 3D vector lengths in the translational setup corrections for all treatment sites are shown in Table 2 and Fig. 4. Three‐dimensional vector distances of ≥ 7 mm were rare for brain lesions and H&N, but more common for T&A and prostate lesions at 25.6% and 12.1%, respectively. In addition, as shown in Fig. 4, the distributions of 3D vector length of the translational shift for brain lesions and H&N decrease rapidly from a starting length of ≥ 1 mm, but these variations for T&A and prostate lesions decline only gradually. The cumulative frequencies of rotational setup corrections for all treatment sites are shown in Table 3 and Fig. 5. In contrast with the 3D vector length of translational shift, these frequencies were all similar regardless of the treatment site. The brain had the highest value with 30.6% of treatment fractions ≥ 1° compared to 17.1%, 19.5%, and 7.9% for H&N, T&A, and prostate lesions, respectively.

**Table 2:** Cumulative frequencies (%) of 3D vector lengths for translational setup error

*3D Vector Length (mm)*	*Brain*	*H&N*	*T&A*	*Prostate*
≥0	100.0	100.0	100.0	100.0
≥1	96.6	98.4	98.7	98.9
≥2	70.5	66.7	91.7	88.5
≥3	31.3	32.6	84.7	71.6
≥4	10.0	9.4	64.9	53.8
≥5	3.9	3.1	50.5	35.9
≥7	0.0	0.4	25.6	12.1
≥10	0.0	0.2	8.0	2.0
≥15	0.0	0.1	0.3	0.5

3D=three‐dimensional; H&N=head and neck; T&A=thorax and abdomen.

**Table 3:** Cumulative frequencies (%) of the magnitudes for rotational setup error

*Magnitude (°)*	*Brain*	*H&N*	*T&A*	*Prosta*
≥0	100	100	100	100
≥1	30.6	17.1	19.5	7.9
≥2	6.4	0.8	3.8	0.0
≥3	0.5	0.0	0.3	0.0

H&N=head and neck; T&A=thorax and abdomen.

The mean setup corrections and SDs for each patient and treatment fraction are shown in 6, 7.

These values indicate the intertreatment setup variations of each patient. The variations were smaller amongst the brain and H&N cohorts than among the T&A and prostate cohorts with respect to the translational directions. In addition, the T&A cohort showed the greatest variations in translational directions. However, the variations in the rotational direction differed from those for the translational directions in some treatment sites. The largest variations were present in the brain cohorts and the lowest were in the prostate cohorts.

The M values for all treatment sites and in all directions are displayed in Table 4. The magnitudes of M for all treatment sites were within ±1 mm and $\pm 0.1^{\circ }$, with the exception of the T&A site, which had overall mean error of 2 mm in the negative longitudinal direction. The Σ and σ for all treatment sites are summarized in Table 5. The largest magnitude of Σ and *a* for the brain lesions and H&N was 1.4 mm in the translational directions, and 3.3 mm for T&A and prostate lesions. In addition, the magnitudes of Σ and σ for all treatment sites were within 0.7° in the rotational direction. Based on these results, the PTV margins were calculated using the van Herk^(40)^ formula and are listed in Table 6. The PTV margins required in this analysis are ≤ 4 mm for the brain lesions and H&N in all translational directions, but ranged from 4 to 10 mm for T&A and prostate lesions.

**Figure 6:**
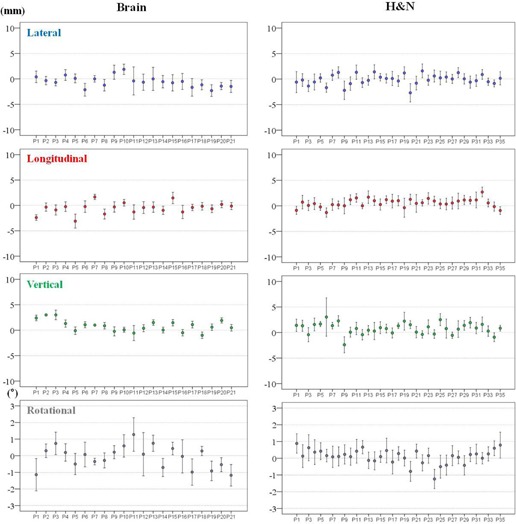
Mean±SD of individual patient setup corrections for brain and H&N sites in all directions. The mean±SD setup corrections indicate intertreatment variations of 21 brain patients and 35 H&N patients. H&N = head and neck, SD = standard deviation.

**Figure 7:**
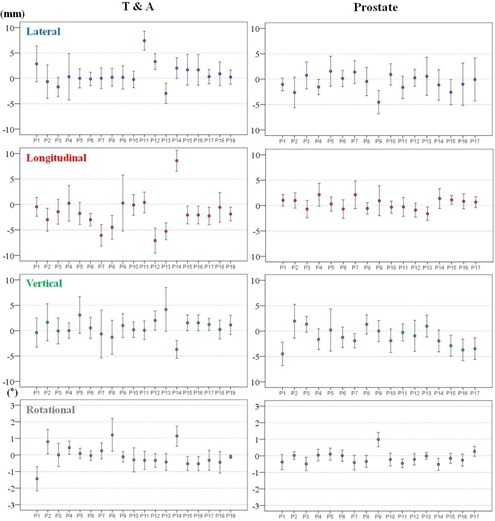
Mean±SD of individual patient setup corrections for T&A and prostate sites in all directions. The mean±SD setup corrections indicate intertreatment variations of 19 T&A patients and 17 prostate patients. T&A = thorax and abdomen, SD = standard deviation.

**Table 4:** The overall mean errors (M) of all directions for each treatment site

	*Lateral y*	*Longitudinal (mm)*	*Vertical (mm)*	*Rotational (°)*
Brain	−0.6	−0.5	0.6	−0.1
H&N	−0.1	0.6	0.9	0.1
T&A	0.5	−2.0	1.0	−0.1
Prostate	−0.6	0.3	−1.0	−0.1

H&N=head and neck; T&A=thorax and abdomen.

**Table 5:** The systematic errors and random errors of all directions for each treatment site

	*Systematic Error* (Σ)				*Random Error* (σ)			
	*Lateral (mm)*	*Longitudinal (mm)*	*Vertical (mm)*	*Rotational (°)*	*Lateral (mm)*	*Longitudinal (mm)*	*Vertical (mm)*	*Rotational (°)*
Brain	1.1	1.1	1.1	0.7	1.4	1.0	0.7	0.7
H&N	1.0	0.8	1.1	0.4	1.2	1.1	1.3	0.6
T&A	2.2	3.3	1.7	0.6	2.5	2.5	2.7	0.6
Prostate	1.6	1.1	1.9	0.4	2.8	1.8	2.4	0.4

H&N=head and neck; T&A=thorax and abdomen.

**Table 6:** The calculated PTV margins for each treatment site using the van Herk^(40)^ formulation.^a^

		*Lateral (mm)*	*Longitudinal (mm)*	*Vertical (mm)*
	Brain	3.73	3.45	3.24
PTV margin	H&N	3.34	2.77	3.66
	T&A	7.25	10.00	6.14
	Prostate	5.96	4.01	6.43

^a^ 2.5Σ+0.7σ

H&N=head and neck; T&A=thorax and abdomen.

## IV. DISCUSSION

In this study, we analyzed the setup uncertainties for the various tumor sites of 92 patients undergoing a total of 2230 fractions by RapidArc. All patients underwent daily CBCT prior to treatment in order to verify setup positioning, target localization, and tumor shrinkage, using an online registration program and an automatic couch system.

The clinical threshold level at our institution is 3 mm and 3° for both translational and rotational directions. In cases of larger deviations, the patient was repositioned and online registration was performed anew.

Intrafractional variations of tumor location and of patient positioning for all treatment sites were not included in our study, because previous studies have reported that this variation was small and that breathing motion was stable.^31^, ^42^ Moreover, Purdie et al.^(43)^ reported that a significant intrafractional variation may occur when the time interval between localization and repeat CBCT imaging exceeds 34 minutes, whereas the total time from localization to treatment in our institution is approximately 15 minutes. In addition, post‐treatment CBCT verification may lead to overexposure of healthy adjacent tissues, skin, bone, lenses, and organs such as the pituitary and thyroid glands.

Previous studies^21^, ^37^, ^44^ have addressed setup uncertainties for brain and H&N treatment sites and described a number of common factors that can contribute to setup errors for these sites. The factors consist of curved external anatomy, a loosening of the fixation mask due to a reduced body contour following weight loss, and tightening of the mask by the swelling of some part of the lesion.

In consideration of these factors, the fixation mask was remade in our institution if considerable discrepancies occurred, and rescanning and replanning were performed to rduce setup errors for these sites. Based on the previous observation^(44)^ that a patient‐specific vacuum bag headrest can reduce setup errors in the vertical direction, all patients with a tumor in the brain or H&N region used a patient‐specific headrest made from rigid urethane compound.

As a result, cumulative frequencies of 3D vector lengths of ≥ 4 mm were only present in 10% and 9.4% of brain and H&N cases, respectively, and the magnitudes of Σ and σ for these sites were small (≤ 1.4 mm). However, the brain site has the broadest distribution and highest magnitude of cumulative frequencies of rotational corrections among all treatment sites. A possible explanation for this could be that the fixation mask for patient with brain tumors in our institution covered only the head, as shown in Fig. 1(a), and these patients might therefore be able to move more easily along the occipito–odontoid axis in the rotational direction than patients with H&N. However, the magnitude of rotational setup errors for the brain was not significant, because only 0.5% of rotational corrections were 3° or greater, and the magnitudes of the M, Σ, and σ did not exceed 0.7°.

Multiple studies have reported on the uncertainty of target localization^14^, ^28^, ^45^, ^46^ and of immobilization systems^14^, ^30^ for T&A using CBCT image guidance. A number of common factors may affect the setup error in T&A treatment, which includes internal respiratory motion, patient movement, tumor location, tumor shrinkage, registration technique, and the use of different immobilization systems.

**Figure 8:**
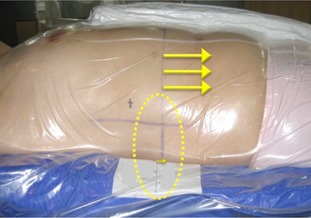
A possible longitudinal setup error for T&A patient using vacuum‐mold system. Patient' skin and external fiducials tend to be pulled in a negative longitudinal direction during the creation of a whole‐body vacuum mold; thus, setup error in the negative longitudinal direction for T&A sites may occur. T&A = thorax and abdomen.

Daily CBCT image guidance was used for all T&A patients to check for daily variations in target size, shape, and location. Volumetric image registration uses not only bony structures as a reference, but also the detectable tumor volume and relevant adjacent anatomical structures. Moreover, BobyFix dual vacuum immobilization system was used in order to minimize patient movement during treatment. The Σ and σ for T&A were relatively small compared with other studies.^37^, ^47^ However, the magnitude of M in the longitudinal direction was 2 mm, and the distribution of translation shift was negatively biased. A possible explanation for this could be the use of the vacuum‐mold system. As seen in Fig. 8, the skin and external fiducials tend to be pulled in a negative longitudinal direction during the creation of a whole‐body vacuum mold and, as a result, setup error in the negative longitudinal direction for T&A sites may occur for each patient over the treatment course. In order to prevent this, the degree of vacuum used in the T&A patient's planning CT should be applied equally in the creation of the vacuum mold in patient setup before treatment. It is also important to check the skin or fiducials of every T&A patient undergoing treatment setup or positioning with the vacuum‐mold system prior to CBCT image acquisition.

Several common factors may affect setup errors for the prostate treatment site — rectal and bladder filling, the use of different immobilization systems, treatment table sag, and the accuracy with which the laser is positioned. In order to assess setup error precisely, the same immobilization device (e.g., a personal knee pillow) was applied to all prostate patients, and quality assurance for the accuracy of the lasers and couch deflection was performed to within a threshold of ±1 mm or $\pm 1^{\circ }$ once a month in our institution. It is important to ensure the geometric accuracy of table sag because the prostate gland is located in the posterior of the body and the table is extended into the linear accelerator, allowing it to be tilted. In addition, the weight of the patient may affect table sag. In order to rduce setup error in the vertical direction, these factors should be reduced as much as possible because variations in rectal and bladder filling can greatly affect prostate position in the vertical direction.^48^, ^49^

Rectal ballooning and bladder filling depends on hw well the patient is instructed. Image registration should be performed strictly based solely on the shape of rectum and bladder, and the position of the prostate gland and pelvic bony anatomy.

The magnitudes of M, Σ, and σ of the prostate sites were small (≤ 2.8 mm), especially the value of the vertical direction of M, which was 1 mm less than previously reported.^(37)^

Multiple studies have reported reduced PTV margins with the use of CBCT image guidance.^14^, ^21^, ^49^ Den et al.^(21)^ demonstrated that the PTV margin without image‐guided radiation therapy should be ≥ 5 mm, whereas with daily CBCT image guidance, it could be reduced to approximately 2‐3 mm, which is a 50% reduction compared to that for the H&N treatment site. The calculated PTV margins of approximately 2.8‐3.7 mm in all translational directions for the brain and H&N treatment sites in our institution were similar to those of Den and colleagues. Therefore, reduced PTV margins for the brain and H&N should be applied under daily CBCT imaging guidance. For T&A treatment sites, the PTV margins were mainly affected by setup errors due to rspiratory motion. Rigid immobilization techniques, such as the double vacuum method (cushion and mold) or the use of a thermoplastic body cast, are practiced in many clinics. These devices can reduce setup uncertainties resulting from the patient's respiration or habitual movement. Another factor that contributes to the PTV margin for the T&A treatment site may be different methods of registration. Yeung et al.^(14)^ demonstrated that discernible soft‐tissue CBCT matching is superior to bony‐tissue matching, which uses bony anatomy as a surrogate for the tumor and can reduce the required PTV margin to 5‐14 mm for lung cancer. CBCT matching was performed without an absolute standard in our institution and the matching was performed differently by considering tumor type, location, shape, extent of motion, and involvement of lymph nodes. As a result, the magnitudes of Σ and σ were reduced to approximately 1‐3 mm, which are similar to those previously reported.^(37)^ As seen in Fig. 7, however, patient‐to‐patient variations and random error in each case during the course of treatment are still inherent to the daily setup. Thus, it is important to be aware that the overall setup errors for T&A sites depend on many institution‐specific variations and, as a result, an appropriate PTV margin should be determined by the analysis of specific setup errors for each clinic using a rigid immobilization device and daily CBCT guidance. The calculated PTV margins for the prostate treatment site were 5.96 mm, 4.01 mm, and 6.43 mm in the lateral, longitudinal, and vertical directions, respectively. These values were larger than the approximate 1‐2 mm recommended PTV margin for prostate treatment in IMRT under CBCT image guidance. A possible explanation for this is that, although rectal and bladder filling was checked by highly experienced therapists and confirmed by the treating physicians, the random movement of each patient throughout the course of treatment is still inherent to the technique, as the immobilization device consists only of a knee pillow. An additional factor is the inclusion of a case which involved a positive pelvic lymph node in our study, which might have enlarged the range of registration and complicated CBCT matching. Therefore, the PTV margin for the prostate site should be adjusted appropriately in each case.

There are a number of limitations that need to be considered with respect to this study. First, the six degrees of freedom (DOF) robotic couch system allows a more accurate evaluation of the setup error, but the system in our institution is only available in four DOF. Although the yaw rotational setup errors for all patients and in all treatment sites were small, pitch and roll rotational setup errors could still arise, and these could not be corrected.

Second, the PTV margins used in the present study are limited because contour and/or deformation errors due to varying tumor locations are not incorporated. However, the clinical data from these errors should be analyzed for further study.

Finally, the intrafractional variation of each patient was ignored in the present study. In order to verify the setup uncertainties more accurately, measurement of intrafractional setup error for specific tumor sites is needed for further statistical analyses.

## IV. CONCLUSIONS

In this study, we analyzed setup errors for various tumor sites treated with a total of 2230 fractions by RapidArc using an automatic couch system and daily CBCT image guidance. We also calculated PTV margins based on the measured systematic and random errors in our institution. Analysis of each institution's specific setup errors using daily CBCT is essential for determining PTV margins, because setup errors vary according to each immobilization system and patient.
